# Small-Molecule Compound CY-158-11 Inhibits Staphylococcus aureus Biofilm Formation

**DOI:** 10.1128/spectrum.00045-23

**Published:** 2023-05-11

**Authors:** Li Shen, Jiao Zhang, Yao Chen, Lulin Rao, Xinyi Wang, Huilin Zhao, Bingjie Wang, Yanghua Xiao, Jingyi Yu, Yanlei Xu, Junhong Shi, Weihua Han, Zengqiang Song, Fangyou Yu

**Affiliations:** a Department of Clinical Laboratory, Shanghai Pulmonary Hospital, School of Medicine, Tongji University, Shanghai, China; b School of Pharmaceutical Sciences, Wenzhou Medical University, Wenzhou, China; c Department of Laboratory Medicine, The First Affiliated Hospital of Wenzhou Medical University, Wenzhou, China; The Ohio State University Division of Biosciences

**Keywords:** *Staphylococcus aureus*, CY-158-11, biofilm, cell adhesion, *icaA*

## Abstract

Staphylococcus aureus is an important human pathogen and brings about many community-acquired, hospital-acquired, and biofilm-associated infections worldwide. It tends to form biofilms, triggering the release of toxins and initiating resistance mechanisms. As a result of the development of S. aureus tolerance to antibiotics, there are few drugs can availably control biofilm-associated infections. In this study, we synthesized a novel small-molecule compound CY-158-11 (C_22_H_14_Cl_2_NO_2_Se_2_) and proved its inhibitory effect on the biofilm formation of S. aureus at a subinhibitory concentration (1/8 MIC). The subinhibitory concentration of CY-158-11 not only did not affect the growth of bacteria but also had no toxicity to A549 cells or G. mellonella. Total biofilm biomass was investigated by crystal violet staining, and the results were confirmed by SYTO 9 and PI staining through confocal laser scanning microscopy. Moreover, CY-158-11 effectively prevented initial attachment and repressed the production of PIA instead of autolysis. RT-qPCR analysis also exhibited significant suppression of the genes involved in biofilm formation. Taken together, CY-158-11 exerted its inhibitory effects against the biofilm formation in S. aureus by inhibiting cell adhesion and the expression of *icaA* related to PIA production.

**IMPORTANCE** Most bacteria exist in the form of biofilms, often strongly adherent to various surfaces, causing bacterial resistance and chronic infections. In general, antibacterial drugs are not effective against biofilms. The small-molecule compound CY-158-11 inhibited the biofilm formation of S. aureus at a subinhibitory concentration. By hindering adhesion and PIA-mediated biofilm formation, CY-158-11 exhibits antibiofilm activity toward S. aureus. These findings point to a novel therapeutic agent for combating intractable S. aureus-biofilm-related infections.

## INTRODUCTION

Staphylococcus aureus is a Gram-positive symbiotic bacteria and opportunistic pathogen, mainly colonizing the skin and mucous membranes in approximately 30% of healthy adults (1). It can cause a variety of metastatic or complex infections, ranging from skin and soft tissue infections to pneumonia, bacteremia, sepsis, endocarditis, osteomyelitis, and necrotizing fasciitis (2). The misuse of antibiotics allows drug-resistant bacteria and resistant genes to rapidly emerge, resulting in S. aureus being resistant to almost all known antibiotics (3). Furthermore, implantable medical devices and catheters, such as joint prostheses, fluid shunts, intravenous catheters, and urinary catheters, are widely used in clinical practice (4). The biofilm formation of S. aureus on biomaterials surfaces is usually persistent and multidrug-resistant, leading to chronic and recurrent infections (5). Biofilm can protect bacteria from the host immune response, antimicrobial agents and/or disinfectants, and various environmental stresses (6). Biofilm-associated infections are a significant cause of morbidity and death, which brings great difficulties for clinical prevention and treatment. The failure of traditional antibiotics in treating biofilm-associated S. aureus infections and the absence of specific antibiofilm agents yet pose a serious threat (7). Consequently, there is an urgent need to develop new drugs that can both effectively inhibit biofilm formation and prevent bacteria from mutating into drug resistance (8).

Bacterial biofilms are defined as a kind of special colony structure that is held together by a self-produced polymer matrix mainly composed of polysaccharides, secreted proteins, and extracellular DNAs (9). The formation of biofilm is a complex, dynamic, and regulated process that involves many factors and can be described in four main phases, including initial attachment, irreversible attachment, maturation, and dispersion (10). Once the initial attachment is completed, extracellular adhesion proteins and cell wall-anchored proteins, such as FnBP and Clf embedded in the peptidoglycan on S. aureus, are constantly secreted. The next stage of biofilm formation is the intercellular aggregation of bacterial cells, which depends on the production of the extracellular matrix (ECM). Polysaccharide intercellular adhesin (PIA) is the main agglutination agent in the biofilm-forming and is encoded by the intercellular adhesion *(ica*) locus (11). Besides, extracellular DNA (eDNA) is also an important component of the biofilm matrix released through a process called autolysis (12). Autolysins are enzymes that degrade the peptidoglycan cell wall layer and are called peptidoglycan hydrolases (13). Atl is the major autolysin in S. aureus, which is regulated by the Cid/Lrg holin-antiholin system (14). Phenol-soluble modulins (PSMs) are key beneficial proteins that can contribute to biofilm dispersal and the spread of planktonic bacteria owing to their surfactant-like properties (15).

CY-158-11 (C_22_H_14_Cl_2_NO_2_Se_2_) is a small molecule compound that contains N-maleimide and diphenyl diselenide (16). Maleimide motifs are found in many natural products, pharmaceuticals, bioactive molecules, and functional materials (17). Meanwhile, it can act as a useful building block for the synthesis of complex products and possess a wide range of biological activities, including antimicrobial, anti-tumor, and anti-inflammatory activity (18). Selenium (Se) is an essential micronutrient for life (19), and the incorporation of Se atoms into small molecules can significantly enhance their bioactivities. Natural and synthetic Se-containing compounds display broad-spectrum potent activities such as anti-cancer, anti-oxidant, anti-parasitic, anti-bacterial, antiviral, anti-fungal, and neuroprotective effects (20). In this study, we investigated how the small-molecule compound CY-158-11 affected the development of S. aureus biofilms and assessed its therapeutic potential for preventing bacterial adhesion and biofilm formation.

## RESULTS

### CY-158-11 inhibits the growth of S. aureus.

**FIG 1:**
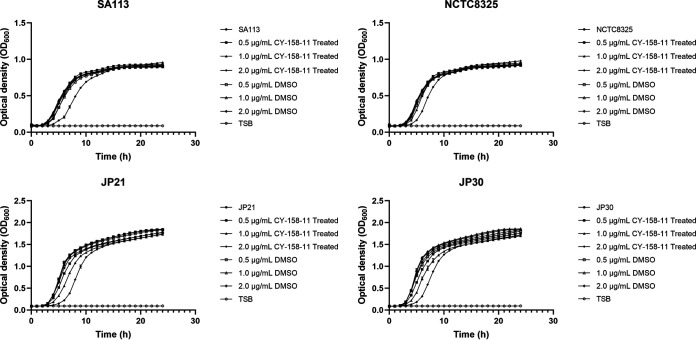
Growth curves of S. aureus strains treated with CY-158-11. Strains were cultured with 0.5, 1.0, and 2.0 μg/mL or without CY-158-11. Trypticase soy broth (TSB) was used as a negative control. DMSO was used as a control to exclude the influence of solvent on bacterial growth. Each test was performed independently in triplicate. The figure shows representative results of one experiment.

The MIC values of CY-158-11 against S. aureus strains SA113, NCTC8325, JP21, and JP30 were all 4 μg/mL. As shown in Fig. 1, the impact of CY-158-11 on the growth of S. aureus was explored by a series of concentrations (0.5, 1.0, and 2.0 μg/mL), indicating that there was no significant difference in the growth of any isolates observed at 0.5 μg/mL CY-158-11. Compared to untreated controls, bacterial growth at concentrations of more than 2.0 μg/mL was altered markedly, and some bacterial growth was impacted at 1.0 μg/mL. Additionally, JP strains also grew more slowly than the other two strains at the subinhibitory concentration of 1.0 μg/mL. We speculated that JP strains may be more susceptible to CY-158-11, possibly due to strain specificity. Thus, CY-158-11 with a subinhibitory concentration of 0.5 μg/mL slightly and transiently inhibited the growth of S. aureus from the initial phase after drug exposure and the planktonic bacteria growth would almost restore the growth capacity in the late logarithmic phase.

### CY-158-11 inhibits the biofilm formation of S. aureus.

**FIG 2:**
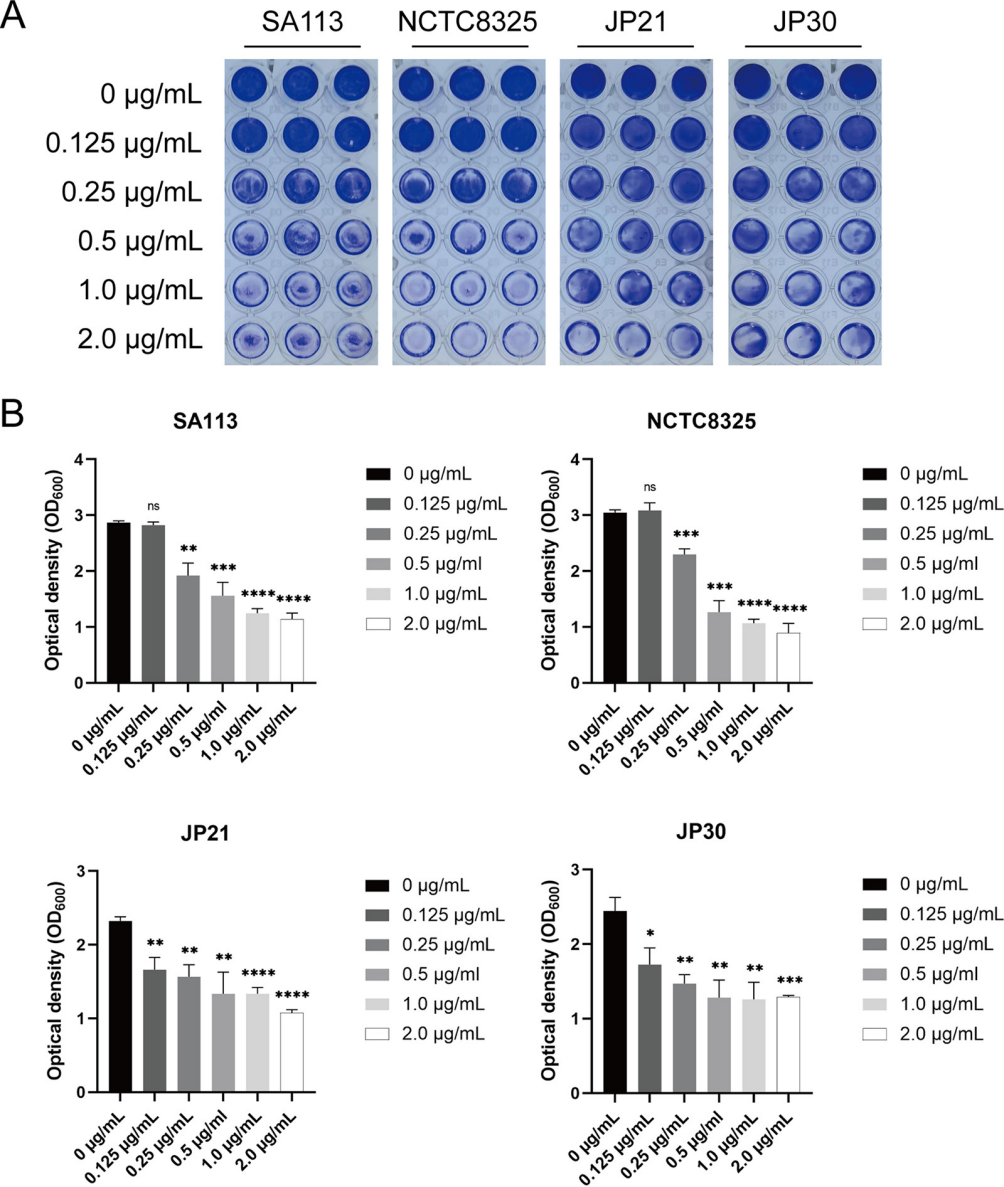
Effect of CY-158-11 on S. aureus biofilm formation. (A) Photographs of crystal violet-stained microtiter plate wells (in triplicate). (B) The optical density (OD) value at 600 nm. *, *P* < 0.05, **, *P* < 0.01; ***, *P* < 0.001, and ****, *P* < 0.0001; ns, no significance. Each test was performed independently in triplicate. The figure shows representative results of one experiment.

The influence of CY-158-11 on the biofilm formation of four S. aureus strains was investigated by various concentrations (0.125, 0.25, 0.5, 1.0, 2.0 μg/mL) using crystal violet staining, demonstrating the distinct inhibition of the biofilm formation of S. aureus (Fig. 2a). Considering that the biofilm formation can be affected by the bacterial growth rate, we selected the subinhibitory concentration of 0.5 μg/mL that didn't affect growth. The biofilms of S. aureus strains treated with CY-158-11 at 0.5 μg/mL were significantly weakened compared to those of the untreated strains, reduced by 45.677 ± 8.402% for SA113, 58.464 ± 6.800% for NCTC8325, 42.547 ± 12.661% for JP21, and 47.551 ± 9.680% for JP30, respectively (Fig. 2b). Therefore, CY-158-11 could inhibit the biofilm-forming ability of S. aureus in a dose-dependent manner. Subsequent discussions would focus exclusively on the effect of CY-158-11 at 0.5 μg/mL.

**FIG 3:**
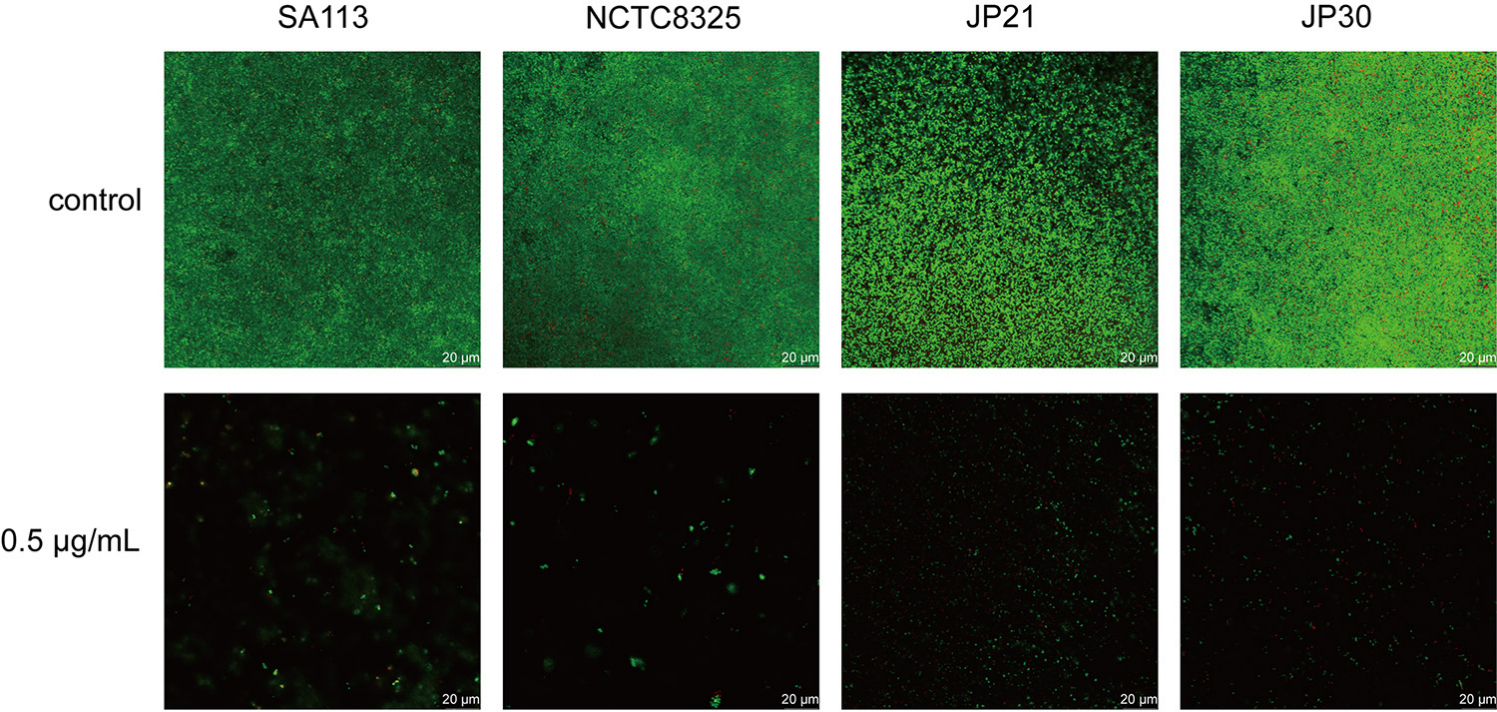
Effect of CY-158-11 on S. aureus biofilm formation observed by CLSM. The biofilms were stained with SYTO 9 and PI: SYTO 9-all cells; PI-cells with permeabilized membranes. Biofilms not exposed to CY-158-11 were used as controls. Scale bar = 20 μm.

Moreover, the effect of CY-158-11 at 0.5 μg/mL on the biofilm formation of S. aureus strains was also evaluated using by confocal laser scanning microscope (CLSM). SYTO 9 green fluorescent nucleic acid stain can stain live and dead Gram-positive and Gram-negative bacteria and PI can selectively embed into DNA double helix in dead cells to give red fluorescence (21). As exhibited in Fig. 3, compared to the fluorescence intensity with the control group (untreated biofilms) for each strain, less green fluorescence occurred in the groups of CY-158-11. More specifically, when growing with CY-158-11, there were only a small number of bacteria evenly distributed in JP21 and JP30, and some scattered small aggregates were observed in SA113 and NCTC8325. These results indicated that the density of the biofilm was notably decreased under the treatment of CY-158-11.

### CY-158-11 inhibits the adhesion of S. aureus.

**FIG 4:**
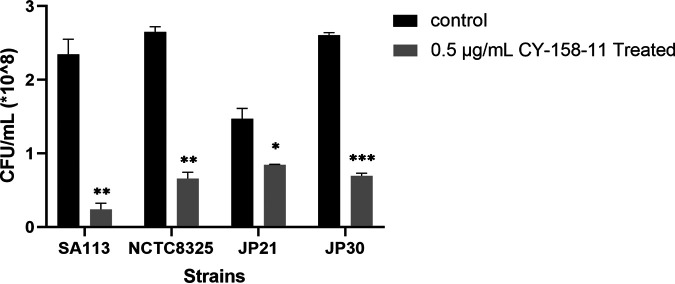
Effect of CY-158-11 on S. aureus cell adhesion. The number of attached cells treated with 0.5 μg/mL CY-158-11 or not by colony counting. *, *P* < 0.05; **, *P* < 0.01; and ***, *P* < 0.001. Each test was performed independently in triplicate. The figure shows representative results of one experiment.

To explore whether the subinhibitory concentration of CY-158-11 had an inhibitory effect on the adhesion of S. aureus, we observed the number of viable bacteria colonies with or without CY-158-11 treatment by colony counting. After the addition of 0.5 μg/mL CY-158-11, the adhesion abilities of four S. aureus strains to the solid surfaces were significantly suppressed by 89.765 ± 3.618% for SA113, 75.094 ± 3.202% for NCTC8325, 42.517 ± 0.481% for JP21, and 73.321 ± 1.357% for JP30, respectively, compared to the untreated controls (Fig. 4). There was an evident trend that the subinhibitory concentration of CY-158-11 could inhibit the biofilm formation at the stage of initial attachment (3 h).

### CY-158-11 inhibits the autolysis ability of S. aureus.

**FIG 5:**
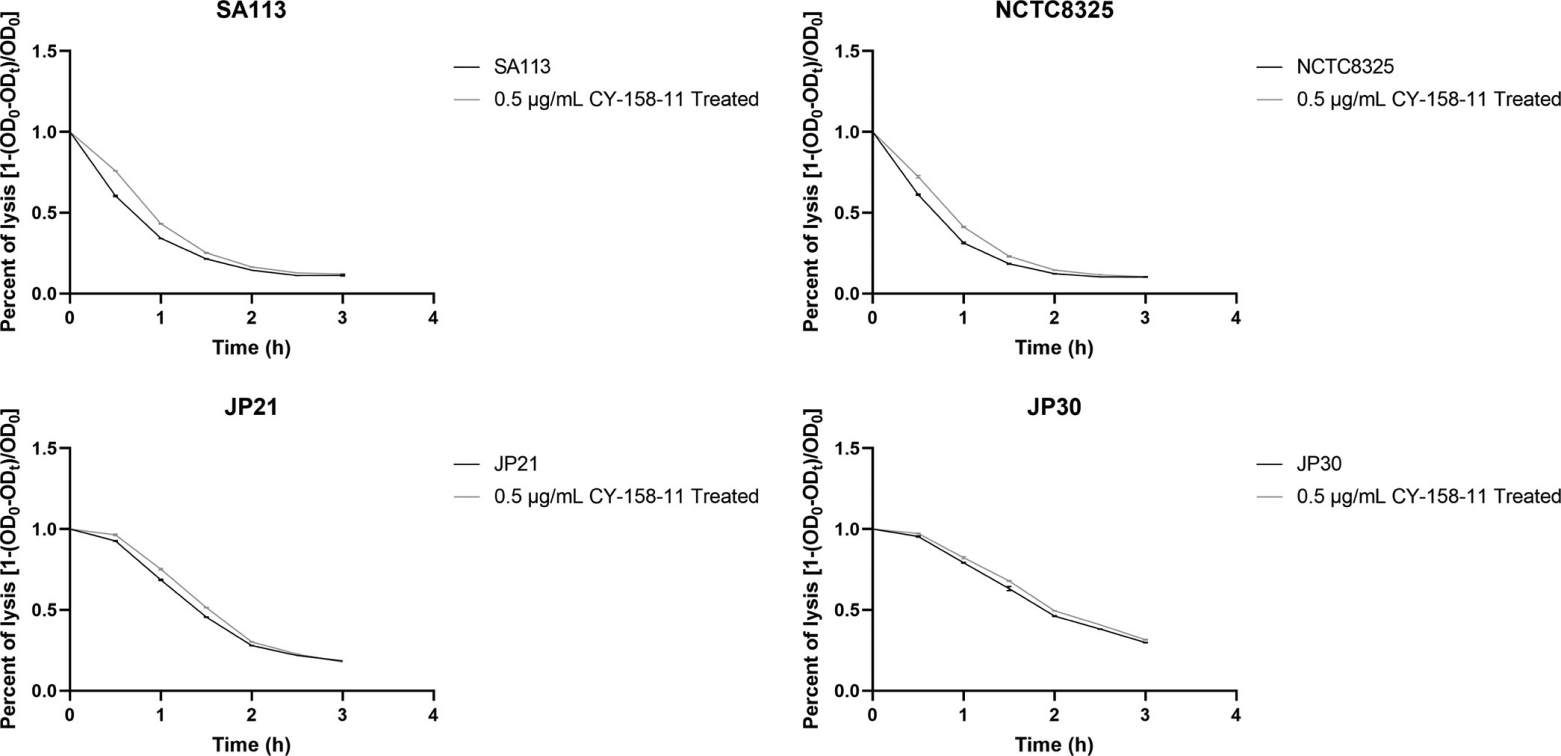
Effect of CY-158-11 on S. aureus autolysis ability. Each test was performed independently in triplicate. The figure shows representative results of one experiment.

The autolysis ability of bacteria can promote the formation of biofilm (22). We used TritonX-100 to induce the autolysis of S. aureus and plotted the autolysis curve. The results showed that the autolysis abilities of four S. aureus strains SA113, NCTC8325, JP21, and JP30 treated with CY-158-11 at a concentration of 0.5 μg/mL were slightly decreased compared with those of the untreated strains (Fig. 5), suggesting that CY-158-11 had little effect on the autolysis ability of S. aureus. Therefore, autolysis was not required for CY-158-11 to limit the formation of S. aureus biofilms.

### CY-158-11 inhibits the production of PIA.

**FIG 6:**
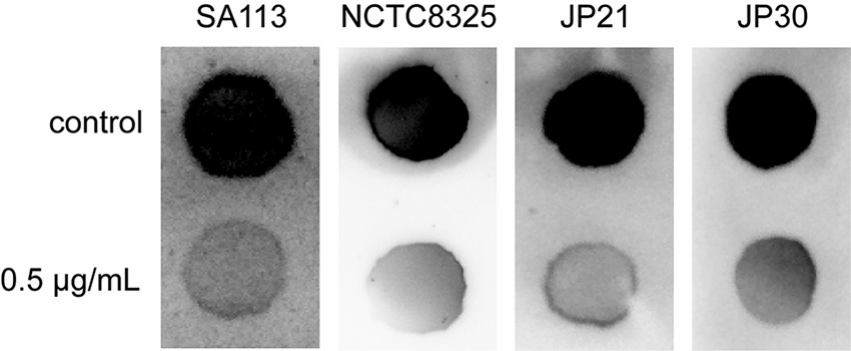
Effect of CY-158-11 on S. aureus PIA production. PIA samples extracted from untreated bacterial cells were used as controls.

The PIA is identified as a major component of the Staphylococcal biofilm (23). The release of polysaccharide intercellular adhesin (PIA) in cultures was determined semiquantitatively to investigate the effect of CY-158-11 on the production of the biofilm matrix. As depicted in Fig. 6, compared to that of the untreated controls after 24h of biofilm formation, the PIA production of four S. aureus strains SA113, NCTC8325, JP21, and JP30 in the presence of 0.5 μg/mL CY-158-11 were substantially reduced. Accordingly, these results suggest the subinhibitory concentration of CY-158-11 achieved the inhibition of S. aureus biofilm formation by reducing the production of PIA.

### CY-158-11 inhibits the expression of biofilm-related genes.

**FIG 7:**
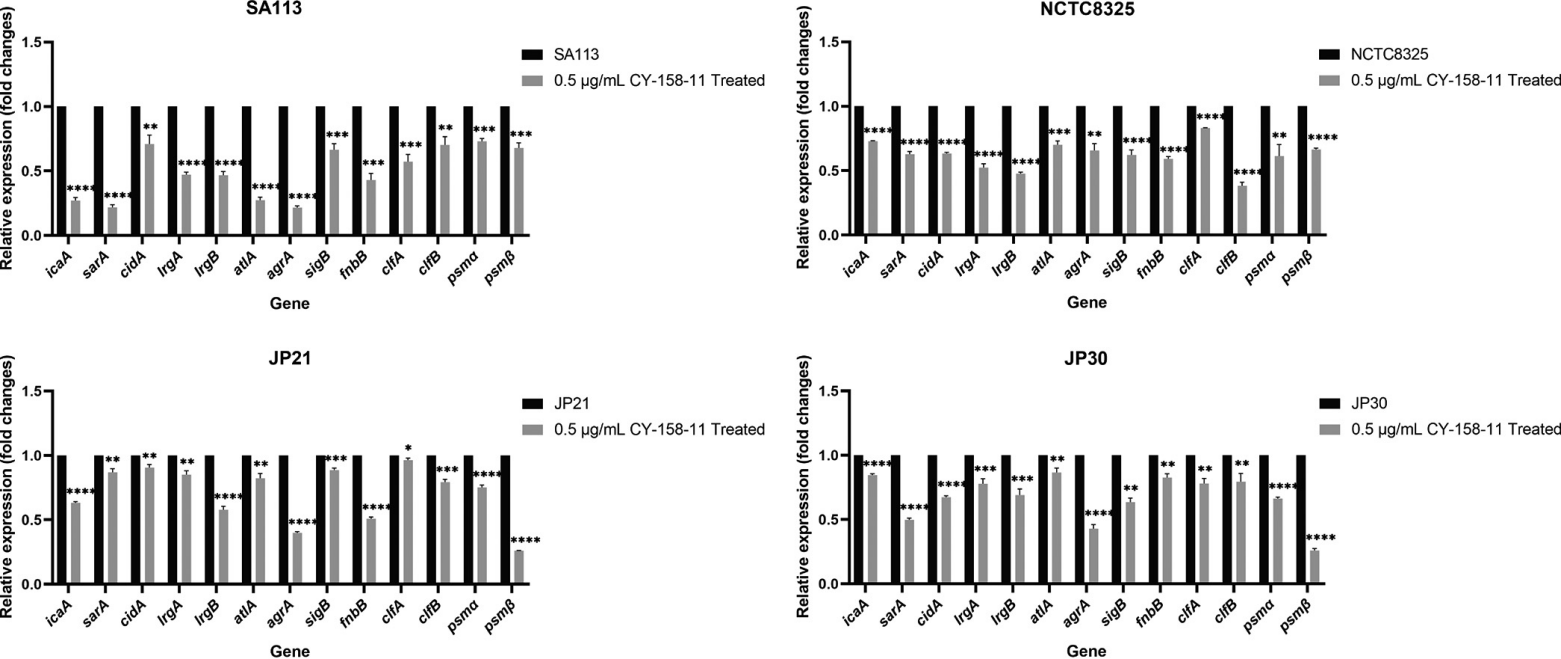
Effect of CY-158-11 on S. aureus biofilm-related genes expression. *, *P* < 0.05; **, *P* < 0.01; ***, *P* < 0.001; and ****, *P* < 0.0001. Each test was performed independently in triplicate. The figure shows representative results of one experiment.

To clarify the mechanism by CY-158-11 suppressed biofilm formation, we compared the transcript levels of biofilm-related genes between CY-158-11 treated and untreated S. aureus strains by RT-PCR. As illustrated in Fig. 7, the expression of *icaA*, *sarA*, *cidA*, *lrgA*, *lrgB*, *atlA*, *agrA*, *sigB*, *fnbB*, *clfA*, *clfB*, *psmα*, and *psmβ* genes was downregulated to various degrees under the treatment of 0.5 μg/mL CY-158-11. These results were consistent with the previous biofilm-related phenotypic assays, which further explained the findings that the adhesion capacity and PIA production of S. aureus could be suppressed by CY-158-11.

### CY-158-11 is nontoxic to A549 cells and G. mellonella larvae.

**FIG 8:**
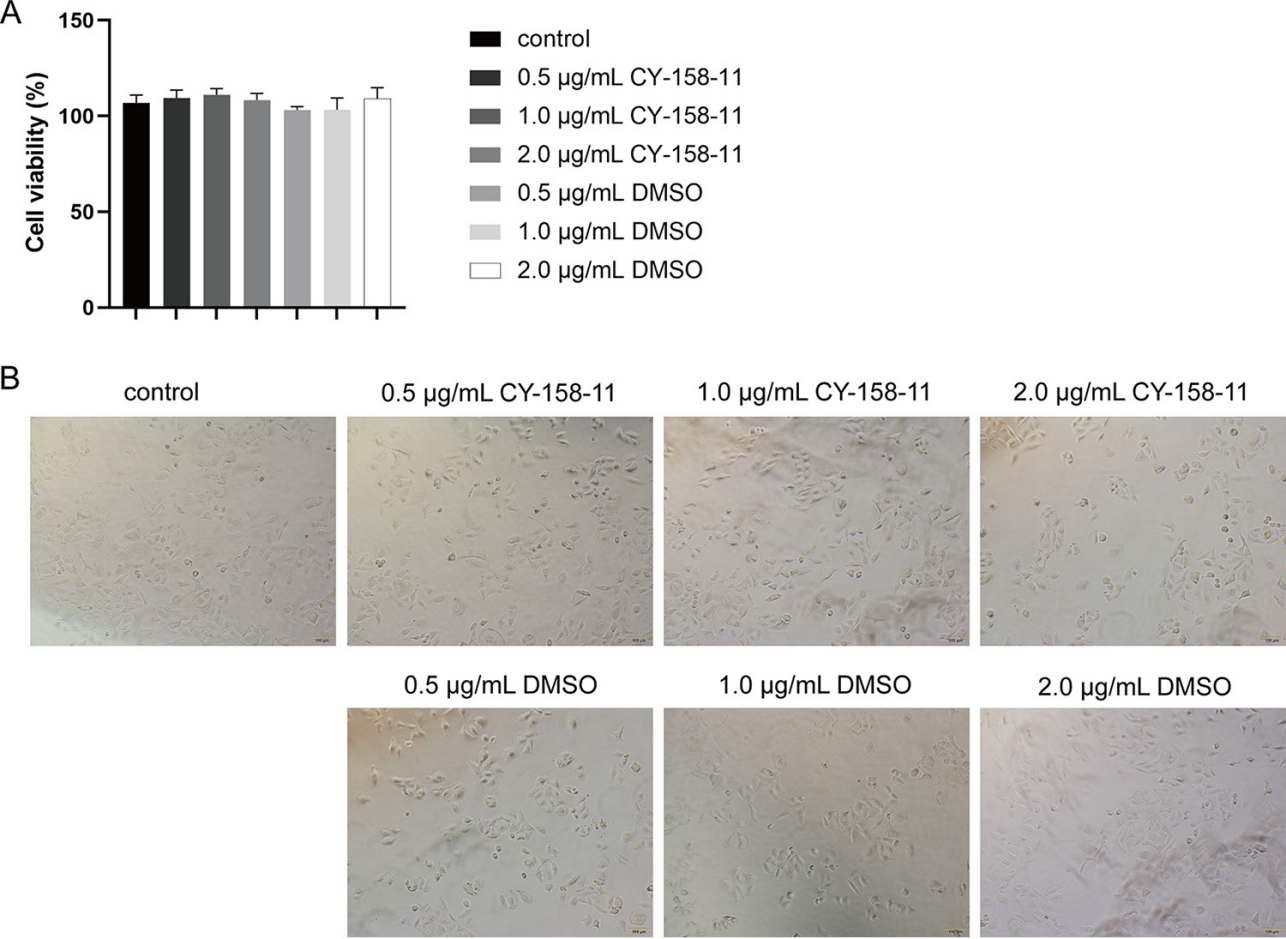
Effect of CY-158-11 on A549 cells. (A) Viability of A549 cells treated with CY-158-11 (0.5, 1.0, and 2.0 μg/mL) or not as determined by CCK-8 assay. (B) Cell morphology and number detected by light microscopy. DMSO was used as a control to exclude the influence of solvent on cytotoxicity. Scale bar = 100 μm.

**FIG 9:**
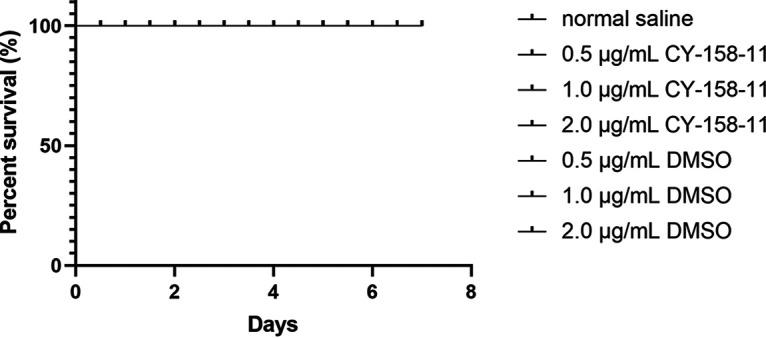
Survival curves of G. mellonella larvae treated with CY-158-11. Larvae were injected with CY-158-11 (0.5, 1.0, and 2.0 μg/mL) or normal saline. DMSO was used as a control to exclude the influence of solvent on larvae survival.

We assessed the cytotoxicity of CY-158-11 with the CCK-8 assay using A549 human alveolar epithelial cells. There were no statistically significant differences in survival rates between the treated groups and untreated groups (Fig. 8a). Observed under an inverted microscope, the cell morphology was normal at 100 μm in all groups (Fig. 8b). Additionally, we evaluated the toxicity of CY-158-11 to G. mellonella larvae by injecting with normal saline or CY-158-11 (0.5, 1.0, and 2.0 μg/mL) and no larva died during the week observed (Fig. 9). The results of these two toxicity tests were consistent, implying that subinhibitory concentrations of CY-158-11 were nontoxic to A549 cells and G. mellonella larvae.

## DISCUSSION

Biofilm is entirely a different entity compared to its planktonic form in terms of biological characteristics, pathogenicity, and immunity. It protects bacteria against environmental stress or the immunological response and is difficult to remove. The ability of microorganisms to form biofilm often leads to the emergence of antibiotic resistance (24). The formation of biofilm, coupled with the dramatic increase in resistance of S. aureus to various antibiotics, remains a severe challenge of clinical reality. Previous studies have shown that subinhibitory concentrations of antibiotics may enhance biofilm formation in multiple bacterial species (25), contributing to poor treatment outcomes. Therefore, it is necessary to transform traditional antibiotics into drugs with new targets that directly inhibit biofilms. For this purpose, we sought a new substance with antibiofilm activity in the present study.

Here, we synthesized a new small-molecule compound CY-158-11, which is a nongrowth-altering biofilm inhibitor. CY-158-11 showed strong antibacterial activity against S. aureus, and its subinhibitory concentrations of ≤ 1/8 MIC did not affect planktonic growth. We hypothesized that CY-158-11 may repress the biofilm formation more significantly with increasing concentration. By establishing an *in vitro* Staphylococcus aureus biofilm model, the biofilm-forming ability test revealed that CY-158-11 effectively prevented the biofilm formation. There was a dose-dependent inhibitory effect of different subinhibitory concentrations on biofilms. Accordingly, we chose the subinhibitory concentration of 1/8 MIC in our experiments to eliminate the possibility that CY-158-11 prevents S. aureus biofilm formation by suppressing the growth of bacteria. We used CLSM to analyze bacterial cell structures at high magnification without damaging the cells (26). The results further demonstrated that S. aureus biofilms grown in CY-158-11 at 1/8MIC were much sparser than those grown in the absence of CY-158-11. During biofilm development, planktonic bacteria adhere to biotic or abiotic surfaces and are encased in an extracellular matrix that confers a high degree of protection (27), finally resulting in a stable, climax community. The first step in biofilm development is the attachment of the organism to human cell surfaces or implanted devices. In our study, although some cells still adhered to the surface in the presence of CY-158-11, the amount was substantially lower than in the untreated group. As a result, CY-158-11 significantly impeded the binding of S. aureus bacterial cells to the solid surfaces.

PIA certainly represents the main mechanism of biofilm formation in S. aureus, but, especially for S. aureus, alternative forms of biofilm that are PIA-independent exist (28). The assumption of the possibility of alternative mechanisms to produce biofilms was shown in research, implying that the inclusion of extracellular polymeric substances was different than PIA, such as eDNA and teichoic acids, in the biofilm matrix (29). Autolysins are closely related to a series of metabolic activities, such as growth, turnover, cell adhesion, cell lysis, and biofilm formation. In S. aureus, the *cid* and *lrg* operons are involved in the coordinated regulatory control of autolysins (AtlA) during biofilm development (30). We found that the biofilm inhibitory effect of CY-158-11 was achieved by inhibiting PIA biosynthesis rather than autolysis ability. The genes necessary for PIA biosynthesis are encoded in the *ica* locus, also requiring the presence of *sarA*, which shows high-affinity binding to the *icaA* promoter region (31). SigB, encoded by *sigB* gene, plays a crucial role in the activation of *sarA* expression during stress conditions by binding to the P3 promoter (32). Besides PIA, a wide variety of extracellular virulence antigens of S. aureus have been reported to be associated with biofilm formation, such as FnBPA, FnBPB, ClfA, ClfB, PSMα, and PSMβ (33). PSM expression depends on the activation of the transcriptional factor *agrA* (34), mostly participating in the control of biofilm dispersal activity (35). The results of RT-PCR were consistent with the above, verifying that CY-158-11 could affect S. aureus biofilms again. In conclusion, the formation of S. aureus biofilms can be inhibited by preventing the adhesion of bacteria and upregulating the expression pathway of the *icaA* in the presence of CY-158-11 during biofilm development. And most importantly, CY-158-11 is nontoxic to A549 human alveolar epithelial cells as well as G. mellonella larvae at subinhibitory concentrations.

However, this study had some limitations. Although our study confirmed that CY-158-11 at subinhibitory concentrations is nontoxic and effective in inhibiting biofilm formation, further research is still required to identify the binding site, action mode, and specific mechanism of this compound inhibition of S. aureus biofilms. S. aureus is a facultative anaerobic bacteria or aerobic bacteria. Anaerobic conditions stimulate PIA production, resulting in enhancing biofilm formation in S. aureus (36). Furthermore, impaired respiration elicits increased cell lysis through increased expression of the AltA murein hydrolase and decreased expression of wall-teichoic acids. The biofilm formation is facilitated by the AtlA-dependent release of cytosolic DNA and proteins, which is governed by the SrrAB TCRS (37). It remains unknown whether the small-molecule compound CY-158-11 could induce biofilm inhibition through respiratory activation. As a corollary, the phenotypes of this compound in an anaerobic environment are worthy of further study in the future. By elucidating the treatment target and mechanism of action of CY-158-11, it may be a promising new drug for the prevention and treatment of biofilm-associated infections in clinical practice.

## MATERIALS AND METHODS

### Synthesis of CY-158-11.

**FIG 10:**
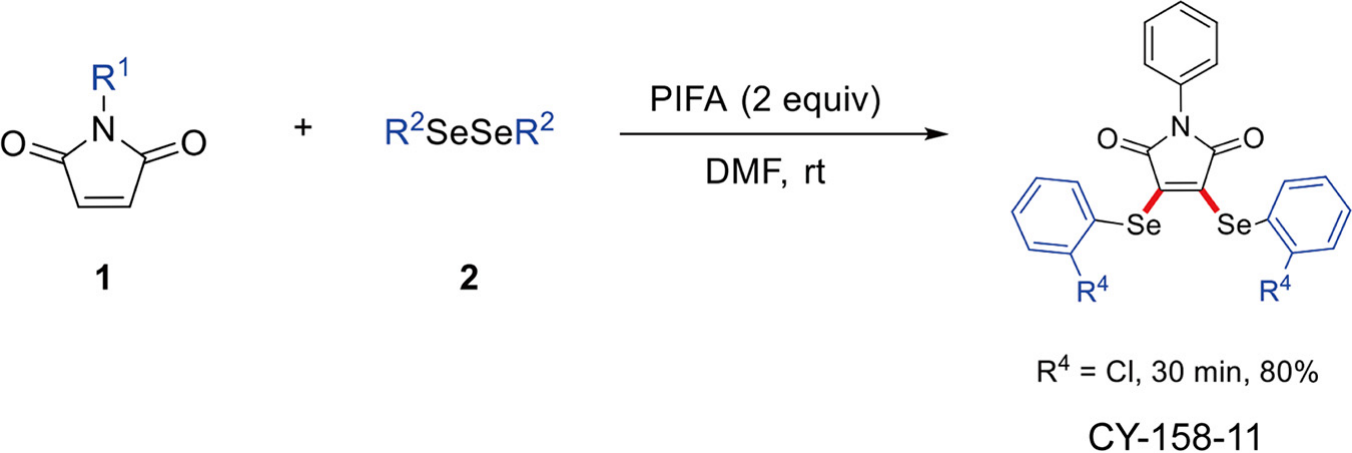
Synthetic process of CY-158-11. 1: Maleimides. 2: Diselenides. CY-158-11: desired product.

A synthesis method of CY-158-11 was performed as described previously (16) (Fig. 10). A mixture of maleimides 1 (0.2 mmol, 1.0 equiv), diselenides 2 (0.3 mmol, 1.5 equiv), and (Bis[trifluoroacetoxy]iodo)benzene (PIFA, 172.0 mg, 0.4 mmol) in N,N-Dimethylformamide (DMF, 2 mL, 0.1 M) was stirred at room temperature for 30 min. After a series of chemical reactions, the desired product CY-158-11 was obtained. The product CY-158-11 (98.052% purity) was obtained as a yellow solid (85.2 mg, 80%). HRMS (ESI) *m/z*: [M+H]^+^ calcd for C_22_H_14_Cl_2_NO_2_Se_2_: 553.8732; found: 553.8724. The full name of CY-158-11 is 3,4-Bis([2-chlorophenyl]selanyl)-1-phenyl-1H-pyrrole-2,5-dione.

### Bacterial strains, cells, and growth conditions.

The strains used in this study were MSSA strains (NCTC8325, SA113, and JP21) and MRSA strains (JP30). NCTC8325 belongs to standard strain, whereas SA113, JP21, and JP30 belong to clinical isolates. SA113 was a gift from the Department of Infectious Diseases and the Key Laboratory of Endogenous Infection, Shenzhen Nanshan People’s Hospital (Shenzhen, China). JP21 and JP30 were isolated from The First Affiliated Hospital of Wenzhou Medical University. The effect of CY-158-11 on the biomass and viability of preformed biofilm was evaluated using these four strong biofilm producer S. aureus strains. We used Trypticase soy broth (TSB, BD Biosciences, Franklin Lakes, NJ, United States) medium without antibiotics to culture all strains at 37°C with shaking at 220 rpm.

The human lung cancer cell line A549 used for the present study is a subpopulation from the American Type Culture Collection (Rockville, MD, USA). A549 cells were maintained in humidified 5% CO2 at 37°C in Dulbecco’s Modified Eagle’s Medium (DMEM, Thermo Fisher Biochemical Products [Beijing] Co., Ltd.) containing 10% fetal bovine serum (FBS, Sigma-Aldrich, St. Louis, MO, United States) and 1% penicillin/streptomycin solution.

### Determination of MIC.

CY-158-11 was dissolved in dimethyl sulfoxide (DMSO, Biosharp, Beijing, China) to a concentration of 16 mg/mL. The MIC of CY-158-11 was determined by broth microdilution method. Bacterial cell suspensions were prepared at 0.5 McFarland standard and diluted 1:100 with cation-adjusted Mueller-Hinton broth (CAMHB). A 100 μL volume of serial dilutions of CY-158-11 and a 100 μL volume of bacteria cell suspensions were added to the 96-well microfilter plate. The MIC was defined as the lowest concentration inhibiting visible growth of bacteria after 16 to 20 h of incubation of the above cultures at 37°C. Three independent trials were performed with duplicate samples.

### Growth assay.

S. aureus isolated colonies were inoculated into tryptic soy broth (TSB) and the suspension was adjusted to a turbidity equivalent to that of a 0.5 McFarland standard. Then bacteria cell suspensions were diluted 1:200 in the tubes containing TSB with subinhibitory concentrations of CY-158-11. The effects of DMSO, whose volume was equal to CY-158-11, on bacteria growth were separately tested. The mixture was divided into three aliquots (200 μL each) and added to a sterile bioscreen honeycomb plate. The optical density at 600 nm every 1 h for 24 h was measured by using an automatic microbial growth curve analyzer (OY Growth Curves, Finland). Ultimately, growth curves were generated from the measured values. The assay was performed in triplicate.

### Biofilm semiquantitative assay.

An overnight culture of S. aureus was diluted 1:100 to the subinhibitory concentrations of CY-158-11 in tryptic soy broth with 0.5% glucose (TSBG) and transferred (200 μL/well) to three parallel wells in 96-well polystyrene microtiter plates. After 24 h of static incubation at 37°C, supernatants were discarded. The wells were washed gently three times with 200 μL of phosphate-buffered saline (PBS, Sangon Biotech [Shanghai] Co., Ltd.) to remove unattached bacteria. The bacterial cells were fixed with 200 μL of methanol for 15 min and stained with 50 μL of 1% crystal violet for 8 min. To remove planktonic cells, running water was used to rinse the excess dye until the water became colorless. After drying, the biofilms were solubilized by adding 30% acetic acid and measured OD_600_ with a microplate reader (Hiwell-Diatek Instruments [Wuxi] Co., Ltd.). Each sample was tested in triplicate.

### Confocal laser scanning microscopy (CLSM).

The biofilm formation was performed in glass bottom cell culture dishes (Biosharp, Beijing, China). An overnight culture of S. aureus was diluted 1:100 with 2 mL TSBG containing 0.5 μg/mL CY-158-11. Strains incubated without CY-158-11 were used as a positive control. After being incubated at stationary phase for 24 h at 37°C to form biofilms, we washed the dishes three times with PBS, and then added 500 μL of SYTO 9 (0.02%, Thermo Fisher Scientific, Waltham, MA, United States) and propidium iodide (0.067%, Thermo Fisher Scientific, Waltham, MA, United States) to stain biofilms for 20 to 30 min at room temperature in the dark. After that, a series of optical sections were scanned by CLSM (TCS SP5; Leica, Wetzlar, Germany) using a 63 × 1.4-numerical aperture oil immersion lens objective.

### Cell adhesion assay.

The S. aureus strains were prepared at the initial attachment phase in the presence and absence of 0.5 μg/mL CY-158-11. Following inoculation at 1:200 into 2 mL TSBG in 6-well plates, the plates were incubated for 3 h at 37°C. After incubation, the wells were washed three times with PBS until fully remove nonadherent and weakly adherent cells, and then resuspended by adding 1 mL PBS. Cell scrapers (BIOLOGIX, Shandong, China) were employed to dislodge those that were firmly attached. Subsequently, bacteria cell suspensions were serially diluted 10-fold and 10 μL of that was plated on TSB agar to determine the number of viable bacteria colonies. Each independent experiment was performed in triplicate.

### Triton X-100-induced autolysis assay.

S. aureus strains were cultured in TSB overnight and added to 50 mL TSB containing 1 M NaCl at 1:200. When the OD_600_ values of the cultures reached 0.3, the cultures were divided into two equal aliquots, of which one was treated with 0.5 μg/mL CY-158-11, and then continuously shaken until the OD_600_ value was 0.7 to 0.8. Bacterial cells were centrifuged at 4,000 g for 20 min at 4°C and washed twice with precooled deionized water. The bacteria were resuspended in a lysis buffer containing 0.05% TritonX-100 (50 mM Tris-Cl, PH = 7.2) and the OD_600_ value was adjusted to 1.0. With shaking at 220 rpm, the optical density at 600 nm was measured every 30 min until 3 h. All experiments were performed in triplicate.

### Enzyme-linked dot immunoblot assay for PIA.

PIA production in S. aureus was detected, with modifications, as described previously (38). Overnight cultures of strains were diluted 1:100 with 3 mL TSBG containing 0.5 μg/mL CY-158-11 or not, respectively, followed by static culture in 6-well plates at 37°C for 24 h. PIA sample was extracted from bacterial cells and spotted onto the nitrocellulose filter membrane. After being dry, the membrane was completely immersed in PBS with 0.1% Tween 20 (PBST, Solarbio, Beijing, China) containing 3.5% bovine serum albumin (BSA, Biosharp, Beijing, China) at 4°C overnight. The semiquantification of PIA was detected by dot blot analysis using wheat germ agglutinin coupled to horseradish peroxidase and visualized via enhanced chemiluminescence (ECL, Beyotime, Shanghai, China).

### RNA isolation and reverse transcription-quantitative PCR.

S. aureus strains were cultured in TSB containing 0.5 μg/mL CY-158-11 at 37°C for 16 h. Positive control was the sterile tubes where bacteria were inoculated without CY-158-11. RNA extraction was performed according to the manufacturer’s instructions (Spin Column Bacteria Total RNA Purification Kit, Sangon Biotech [Shanghai] Co., Ltd.). Next, extracted RNA (1 μg of each) was used as the template for cDNA synthesis by using a PrimeScript RT reagent kit with gDNA Eraser (TaKaRa, Tokyo, Japan). Quantitative real-time PCR (qPCR) was performed by using the TB Green Premix EX taq (Tli RNaseH Plus) (TaKaRa, Tokyo, Japan) and QuantStudio 5 Applied Biosystems (ABI) Fluorescence quantitative PCR instrument (Thermo Fisher Scientific). The primer pairs used for qPCR are shown in Table 1, with *gyrB* as the endogenous gene. Based on a comparative threshold cycle (Ct) method with the formula (2^-ΔΔCt^), fold changes in gene expression were calculated and RNA transcription levels of biofilm-related genes were obtained. Three biological replicates and three technical replicates were performed for each gene tested.

**TABLE 1:** Primers used in this study

Primer	Sequence (5′–3′)
*gyrB*-RT-F	ACATTACAGCAGCGTATTAG
*gyrB*-RT-R	CTCATAGTGATAGGAGTCTTCT
*icaA*-RT-F	GTTGGTATCCGACAGTATA
*icaA*-RT-R	CACCTTTCTTACGTTTTAATG
*codY*-RT-F	GACAATGTATTAACAGTATTCC
*codY*-RT-R	TAGCAGCATATTCACCTA
*sarA*-RT-F	CTTGTGGTTGTTTGTAGTTT
*sarA*-RT-R	GTTATCAATGGTCACTTATGC
*agrA*-RT-F	GCAGTAATTCAGTGTATGTTCA
*agrA*-RT-R	TATGGCGATTGACGACAA
*sigB*-RT-F	TTCCATTGCTTCTAACACTT
*sigB*-RT-R	GATGAACTAACCGCTGAAT
*atlA*-RT-F	GGTGACACTCGTGCTAAT
*atlA*-RT-R	AGGGCATGTGAGATAAGA
*cidA*-RT-F	TCATTCATAAGCGTCTACA
*cidA*-RT-R	TCTTCATACCGTCAGTTG
*lrgA*-RT-F	CTGGTGCTGTTAAGTTAG
*lrgA*-RT-R	GTATTGTTGAGACGATTATTAG
*lrgB*-RT-F	CATCGGAGGTATTGGTATCG
*lrgB*-RT-R	GTAGTTGCTGCTTGAGGTAA
*clfA*-RT-F	CAGCGATTCAGAATCAGA
*clfA*-RT-R	GGCGGAACTACATTATTG
*clfB*-RT-F	CTGAGTCACTGTCTGAATC
*clfB*-RT-R	CTCAGACAGCGATTCAGA
*fnbA*-RT-F	TTCCTTAACTACCTCTTCT
*fnbA*-RT-R	CAATCATATAACGCAACAG
*fnbB*-RT-F	GCGAAGTTTCTACTTTTG
*fnbB*-RT-R	CAACCATCACAATCAACA
*psmα*-RT-F	ATGGAATTCGTAGCAAAATTATTC
*psmα*-RT-R	TAGTTGTTACCTAAAAATTTACC
*psmβ*-RT-F	CCTAGTAAACCCACACCG
*psmβ*-RT-R	GCTGCACAACAACATGATA

### Assessment of CY-158-11 cytotoxicity.

The Cell Counting kit-8 (CCK-8) was used to analyze the cytotoxicity of CY-158-11 to A549 human alveolar epithelial cells. To exclude the influence of the solvent on cytotoxicity, we used DMSO as a control. A549 cells were seeded into 96-well microfilter plates at 10^4^ cells per well. After incubation for 12 h., DMEM containing 10% fetal bovine serum and 1% penicillin/streptomycin solution with CY-158-11, DMSO, or neither were added to the wells (200 μL/well). A549 cells not exposed to CY-158-11 were used as an untreated control. After 24 h, we discarded the supernatant and washed the wells with PBS. Subsequently, 100 μL of serum-free DMEM medium and 10 μL of CCK-8 reagent were added to the wells (110 μL/well), followed by incubation for 1 h and measurement of OD_450_.

### Evaluation of CY-158-11 toxicity to Galleria mellonella larvae.

G. mellonella larvae, also known as wax worm, were injected with 10 μL of a various concentration of CY-158-11 (0.5, 1.0, and 2.0 μg/mL; *n* = ~10 per group). In other words, CY-158-11 was tested by inoculating doses of 0.015 mg/kg, 0.03 mg/kg, and 0.06 mg/kg, respectively. Larvae injected with normal saline were used as negative controls. An equivalent volume of DMSO was also tested to ensure that the solvent did not affect the survival of the larvae. After injection, all the larvae were placed in a clean petri dish and incubated at 37°C for 7 days. The viability of the larvae was observed and recorded at 12 h intervals.

### Statistical analysis.

Statistical analysis was performed by Prism 9.0 software (GraphPad, San Diego, CA). Data were analyzed with Student's *t* test and one-way analysis of variance (ANOVA), which were presented as arithmetic mean ± standard deviations (mean ± SD). *P* values < 0.05 were considered statistically significant. *P < 0.05, **P < 0.01, ***P < 0.001, and ****P < 0.0001.
