# Correction for Shen et al., “Small-Molecule Compound CY-158-11 Inhibits *Staphylococcus aureus* Biofilm Formation”

**DOI:** 10.1128/spectrum.01984-24

**Published:** 2024-08-29

**Authors:** Li Shen, Jiao Zhang, Yao Chen, Lulin Rao, Xinyi Wang, Huilin Zhao, Bingjie Wang, Yanghua Xiao, Jingyi Yu, Yanlei Xu, Junhong Shi, Weihua Han, Zengqiang Song, Fangyou Yu

## AUTHOR CORRECTION

Volume 11, no. 3, e00045-23, 2023, https://doi.org/10.1128/spectrum.00045-23. The sequence for primer *atlA-*RT-F should read "TATGGCTCTGTGAATGGTAA," and the sequence for primer *atlA*-RT-R should read "GGCTTAGGTGTTGGTGTA." We apologize for the error, which did not change the results of the paper.

